# Exploring the Landscape of Operating Room Scheduling: A Bibliometric Analysis of Recent Advancements and Future Prospects

**DOI:** 10.1177/11795972241271549

**Published:** 2025-08-16

**Authors:** Md Al Amin, Majed Hadid, Adel Elomri, Rabah Ismaen, Ismail Dergaa, Hind Alashi, Amal Jobran Al-Hajaji, Moustafa Alkhalil, Omar M Aboumarzouk, Abdelfatteh EL Omri

**Affiliations:** 1 Division of Engineering Management and Decision Sciences, College of Science and Engineering, Hamad Bin Khalifa University, Qatar Foundation, Doha, Qatar; 2 Surgical Research Section, Department of Surgery, Hamad Medical Corporation, Doha, Qatar; 3 Primary Health Care Corporation (PHCC), Doha, Qatar; 4 Corporate Perioperative Nursing, Hamad Medical Corporation, Doha, Qatar; 5 Department of Oral and Cranio-Maxillofacial Surgery, Hamad Medical Corporation, Doha, Qatar; 6 Department of Clinical Science, College of Medicine, Qatar University, Doha, Qatar; 7 School of Medicine, Dentistry and Nursing, The University of Glasgow, Glasgow, UK; 8 Vice President for Medical and Health Sciences Office, QU-Health, Qatar University, Doha, Qatar

**Keywords:** Healthcare management, optimization, patient outcomes, resource allocation, surgical procedures, value-based care, workflow efficiency

## Abstract

**Background:**

Operating Room Scheduling (ORS) is vital in healthcare management, impacting patient outcomes, economics, and the shift to value-based care. The academic literature offers various solutions with distinct pros and cons.

**Aim:**

This study aims to (i) outline ORS challenges across surgical specialties; (ii) examine ORS’s impact on healthcare goals, focusing on patient outcomes, value-based care, and economics; (iii) assess academic solutions’ real-world applicability; and (iv) conduct a bibliometric analysis to track ORS research progression, pivotal works, and future directions.

**Methods:**

We performed a comprehensive bibliometric analysis using Scopus data. Biblioshiny from Bibliometrix aided data mining and analysis, spanning 2000 to 2023, tracking publication trends, themes, co-occurrence, and co-citation networks.

**Results:**

ORS publications steadily rose, notably post-2013, led by developed nations like the UK, Australia, the US, France, and Germany. Key themes included operating rooms, surgery, and humans. Seven primary research routes emerged, covering Surgery Duration, Allocation, Advanced Scheduling Integration, and Patient Flow Optimization. Citation analysis highlighted heuristic algorithms and integer programing as central ORS themes.

**Conclusion:**

This study offers a panoramic ORS overview, advocating an integrated approach aligning patient outcomes, economics, and value-based care. Bibliometric analysis charts ORS research evolution guides future research, and holds significance for practitioners, policymakers, and academics, enhancing ORS paradigms and healthcare delivery.

## Introduction

Operating Room (OR) scheduling is a complex and multifaceted process that plays a crucial role in the efficient functioning of healthcare facilities. Effective OR scheduling ensures the optimal utilization of resources, reduces patient wait times, and enhances the overall quality of care.^1^ However, despite their significance, many healthcare institutions face challenges in creating and maintaining efficient OR schedules.

Several factors contribute to the complexity of the OR scheduling. The unpredictability of surgical duration, variability in surgeons’ preferences, and the need for coordination with other hospital departments make it a challenging task.^2^ Moreover, the dynamic nature of healthcare with emergency cases and last-minute changes adds another layer of complexity.^3^ Recent technological advancements have introduced innovative solutions to address these challenges. Artificial Intelligence (AI) and Machine Learning (ML) algorithms, for instance, are employed to predict surgical durations, optimize resource allocation, and enhance the overall efficiency of OR scheduling processes.^4^ These technologies, combined with data analytics, have the potential to revolutionize OR scheduling. However, although these technological solutions offer promise, their effective implementation requires a comprehensive understanding of the intricacies of OR operations and the challenges faced by healthcare professionals. It is not just about integrating technology but ensuring that it aligns with the real-world needs and constraints of the healthcare environment.^4,5^

The complexities of OR scheduling are further magnified by the increasing number of surgical specialties and the diverse requirements they bring to the table. Each specialty, be it orthopedics, cardiology, or neurosurgery, has its own set of prerequisites, equipment needs, and time considerations.^1^ This diversity necessitates a flexible and adaptable scheduling system that can accommodate the unique demands of each department while ensuring the optimal utilization of the OR.

Moreover, the patient’s journey from pre-operative assessments to post-operative care is intertwined with the OR schedule. Delays or inefficiencies in the OR can have cascading effects impacting patient satisfaction, recovery times, and overall hospital throughput.^2^ Hence, the stakes in OR scheduling are operational and deeply clinical, with direct implications for patient care and safety.

In recent years, the global push toward value-based healthcare has further underscored the importance of efficient OR management. Hospitals are increasingly being evaluated based on outcomes rather than volume, making it imperative to ensure that the OR functions seamlessly and delivers the best possible care to patients. This shift in focus has also brought the economic implications of OR scheduling to the fore, with inefficient schedules leading to significant financial losses for hospitals.

The vast body of literature on OR scheduling reflects the importance and complexity of this topic. Researchers have proposed several models, algorithms, and frameworks to address OR scheduling challenges.^4^ From deterministic to stochastic models and from linear programing to heuristic approaches, the academic community has left no stone unturned in its quest for the optimal OR schedule. However, the dynamic nature of healthcare delivery coupled with the ever-evolving technological landscape means that OR scheduling remains a vibrant and active area of research.

In recent years, bibliometric analysis has emerged as a powerful tool for understanding the academic research landscape. By analyzing published works’ volume, authors, and citation patterns, bibliometric analysis provides insights into the evolution and current state of a particular field of study. In the context of OR scheduling, a bibliometric analysis can offer a comprehensive overview of the most influential works, authors, and trends, thereby guiding future research. Bibliometric analysis is a powerful tool for navigating this extensive body of literature. By systematically mapping the research landscape, we can identify patterns, the evolution of ideas, and pinpoint areas that warrant further exploration.^5^ Such an analysis provides a bird’s-eye view of the field and helps guide future research.^6^

This research responds to a few basic Research Questions (RQs) within each step. The different questions are given separately for ease of presentation. However, their connection is evident, and it is best to consider them as distinct issues rather than separate problems because they ultimately come together to accomplish a common objective.

RQ1: How has the operating room scheduling advanced and its historical development?
RQ2: What are the main research areas and applications?
RQ3: What are the optimization models and solution techniques used for ORS problems?
RQ4: What are the possible pathways and directions for future research?

Based on the complexities and intricacies of OR scheduling, coupled with the evolving landscape of healthcare delivery and the pressing need for efficient, patient-centric solutions, the aims of this study were as follows: (i) To provide a comprehensive overview of the challenges and intricacies inherent to OR scheduling across diverse surgical specialties. (ii) To explore the relationship between OR scheduling and overarching healthcare objectives, emphasizing patient outcomes, transition to value-based care, and economic implications of scheduling decisions. (iii) To critically assess the solutions presented in the academic literature and gage their real-world applicability and effectiveness in various healthcare settings. (iv) To undertake a rigorous bibliometric analysis to chart the progression of OR scheduling research over time, spotlighting seminal works, key milestones, and potential future directions.

## Methodology

**Figure 1.**
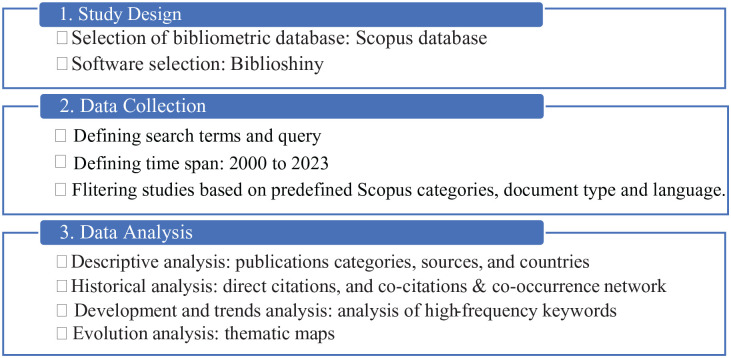
Flowchart of the bibliometric analysis methodology for ORS research.

A bibliometric approach was utilized to evaluate the knowledge base and new breakthroughs in ORS research. The papers under consideration cover topics such as operational management, operational research, and healthcare science services. Articles analyzing ORS from management science, such as health policies, human resources, and capacity, as well as in-depth publications describing health policy and service issues, were included. The methodology employed in this study comprised 5 steps: (1) research design, (2) search terms and queries, (3) data collection and analysis, (4) results and discussion, and (5) suggestions for future research directions. In Figure 1, we provide an overview of the methodology employed in this study.

### Study design

In the first phase, an online bibliographic database and software tools for bibliometric analysis were selected. After analyzing pertinent databases, Scopus was chosen as the source of bibliographic information. The Scopus database is known to have the most extensive collection of scientific research data; as a result, it is among the most commonly utilized databases in bibliometric analyses.^7^ The second stage is a review of the software tools available for bibliometric analysis. Owing to its dependability on handling and merging bibliographic data, an open-source web-based graphical interface for bibliometric analysis called Biblioshiny was used in this study. It connects to other R packages and offers evidence-based quantitative insights into the field under study.^5,8^

### Data collection

In the subsequent stage, the search query terms and Boolean operators were identified to gather the most relevant papers for a comprehensive and in-depth review of ORS research. To accomplish this, a scientific approach was used to identify search terms and criteria, as suggested by Chabowski et al^9^ and Zupic.^10^ The first set of search terms was derived from the relevant literature. Subsequently, a team of experts in the ORS research field was consulted to validate the search query criteria, review the compiled search terms, identify related terms, and add new terms to the list as necessary. The initial set of papers was collected using these search terms and the commonly used keywords used in these papers were identified. Relevant keywords were then added to the list of search terms to expand the search further. This process was repeated several times to obtain the most relevant search terms. A final list of search terms was used for the search query.

After iterative refinement, the search query documented in Table 1 and the criteria for inclusion and exclusion outlined in Figure 1 were employed. The methodology adhered to the Preferred Reporting Items for Systematic Reviews and Meta-Analyses (PRISMA) 2020 statement and checklist as well as guidelines for assessing the methodological quality of systematic reviews: A Measurement Tool to Assess Systematic Reviews (AMSTAR 2).

**Table 1.** Search query and refining criteria.

Database	Scopus
**Search query**	(optimi?* or plan* or manag* or operat* or schedul*) AND (operat* room or operat* theat*)
**Search within**	Article’s title, abstract, and keywords
**Document type**	Article, conference paper, book
**Language**	English
**Scopus Categories**	Engineering, computer science, mathematics, decision sciences, multidisciplinary
**Time Period**	2000-2023

**Figure 2.**
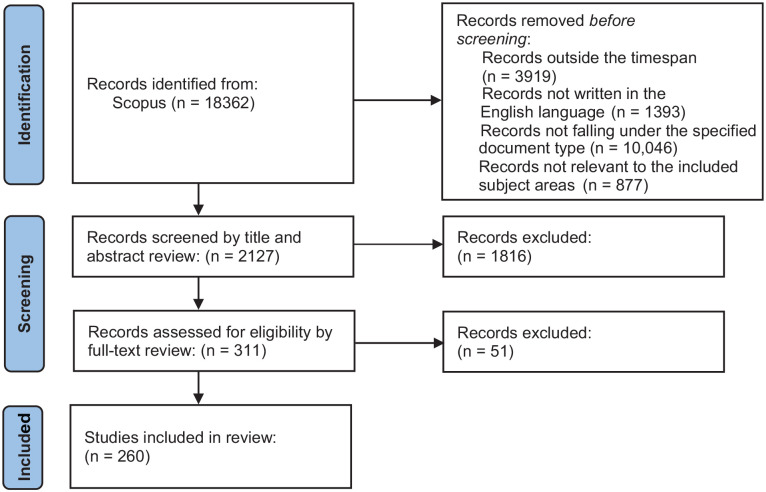
Flow chart of the study selection process based on the systematic reviews and meta-analyses (PRISMA) protocol.

Figure 2 illustrates the document collection process. An initial search of the Scopus database was conducted using the search terms outlined in Table 1, which yielded 18 362 documents. Scopus database was selected for its authoritative and exhaustive collection of peer-reviewed literature, encompassing a broad spectrum of scientific disciplines and consistently maintaining high-quality source material particularly for research in decision sciences and healthcare. To focus on the analysis of recent developments in the field, the time span was initially unrestricted. However, after applying the necessary filters and limiting the search to the years 2000 to 2023, the dataset was refined to 14 443 documents. To further enhance the relevance of the dataset, a 20-year timeframe was chosen. Subsequently, filtering the documents in English resulted in a dataset of 13 050 documents. Additional refinement by document type reduced this number to 3004.

To ensure precision, literature reviews pertaining to ORS were excluded, as they often reference numerous articles that can influence network creation and keyword analysis. Further narrowing was achieved by including specific subject areas, which yielded a dataset of 2127 documents. This dataset was then screened by abstract and title, reducing it to 311 documents. Finally, a detailed examination of the full text was conducted to ensure the inclusion of only the relevant papers. A total of 260 documents were selected for analysis, authored by 899 researchers from various countries and originating from 164 different sources.

We conducted a snowballing backward and forward reference search on the 260 documents to ensure maximum coverage and minimize the risk of missing relevant papers.

### Data Analysis

The data from the Scopus database underwent a comprehensive network analysis to delve deeper into ORS research. This work aims to discover recent advancements and future prospects by performing a thorough bibliometric analysis using R-tools, which provides a wide range of statistical and graphical analyses. For this purpose, the Biblioshiny software developed by Aria and Cuccurullo^5^ was used. Biblioshiny not only facilitates a general statistical analysis of articles but also illuminates the pivotal aspects of a research domain.^11^ Through this analysis, we identified the core areas of ORS research, the predominant categories, the literature’s leading contributors, and nations with the highest productivity. This descriptive bibliometric analysis revealed the diverse research topics encompassed by ORS. Notably, the Scopus database predominantly features healthcare science services, including health policy services, healthcare management, and operations research related to operating room scheduling. Consequently, we narrowed our document search to fields such as engineering, computer science and engineering, mathematics, decision sciences, business, management and accounting, health, nursing, and multidisciplinary research.

## Results and Discussion

### Descriptive analysis of ORS publications

#### Annual production and average citations per year

**Figure 3.**
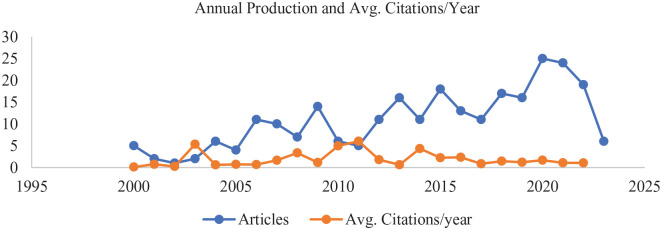
Annual publication trends and average citations for ORS research (2000-2023).

Figure 3 presents the statistics for the ORS publications based on the Scopus database. This figure illustrates the yearly publication count from 2000 to 2023 and the cumulative citations for the 260 articles chosen for this study. The data indicate a marked surge in ORS publications post-2013, although the average yearly citations have seen a decline. Between 2008 and 2012, the annual publication counts in this domain remained relatively consistent, barring a noticeable dip in 2010 and then experiencing a sharp uptick from 2013 to 2023.

As this study underwent multiple iterations, adjusting search terms and time spans, it was observed that the publications from 2008 to 2023, essentially the last 15 years, predominantly centered on topics such as operating scheduling and optimization,^12-24^ multi-objective optimization,^25-33^ optimization under uncertainty,^34-36^ ML in operating room scheduling,^37-40^ heuristics and simulation in operating room scheduling,^23,41-45^ and multi-criteria decision-making methods tailored to the operating room scheduling problem.^46-48^

#### Research by source and countries

**Figure 4.**
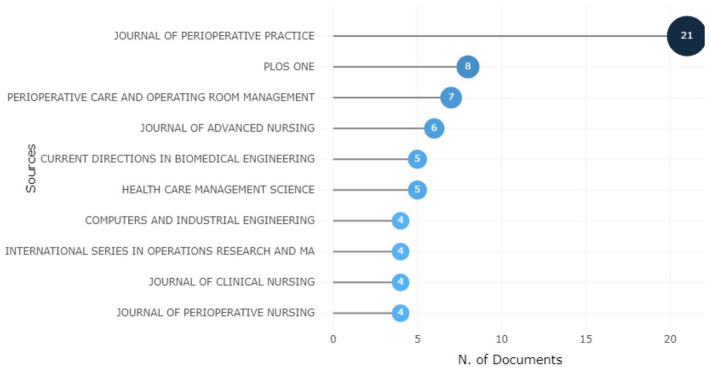
Primary sources of ORS research publications.

This study employed a systematic search strategy to identify relevant sources for the analysis of operating room scheduling and optimization. As depicted in Figure 4, the primary sources included journals such as perioperative practice, PLOS, perioperative care, and operating room management. Furthermore, core sources in the field, such as Computer and Industrial Engineering, Operations Research, and renowned journals, such as Healthcare Management Science, Journal of Medical Systems, and Journal of Clinical Nursing, were considered. Moreover, lecture notes and survey reports on ORS were also included as sources.

**Figure 5.**
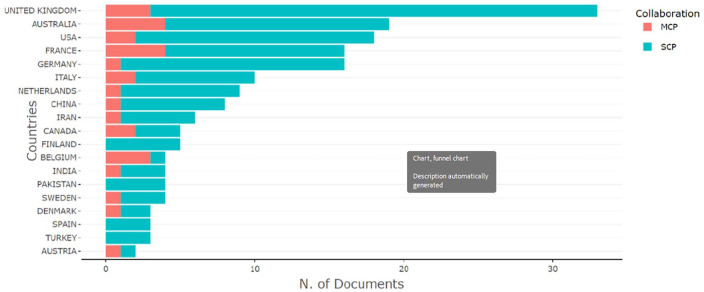
Geographical distribution of ORS-related publications (2000-2023). Abbreviations: MCP, Multi Country Publication; SCP, Single Country Publication.

Figure 5 illustrates the distribution of ORS-related publications across countries based on the Scopus database. The analysis revealed that the United Kingdom, Australia, the United States, France, and Germany were the top 5 countries in terms of ORS-related publications. Most countries have engaged in collaborative research on ORS, with France and Australia being the most active collaborators. However, some countries, including Finland, Pakistan, Spain, and Turkey, have not undertaken any collaborative research. Austria exhibited the least research output on ORS among all countries during the period from 2000 to 2023. Between 2008 and 2023, all countries performed collaborative research on ORS, except Jordan and the Netherlands. Furthermore, Indonesia, Korea, and Morocco have not yet engaged in collaborative research on ORS during this period, and New Zealand has exhibited the lowest research output among all countries. These findings provide insights into the global landscape of ORS research and may guide future collaborative efforts to advance this field.

### Historical analysis of ORS research field

#### Direct citations and collaborative networks

**Figure 6.**
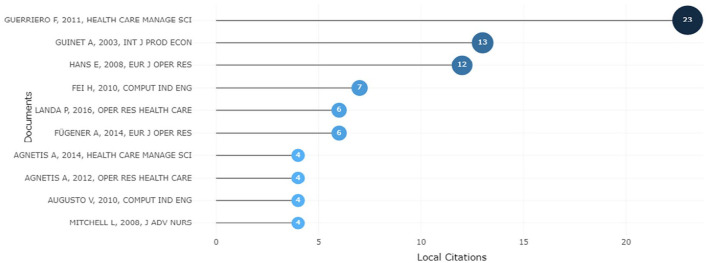
Top 10 locally cited ORS documents from 2000 to 2023.

**Figure 7.**
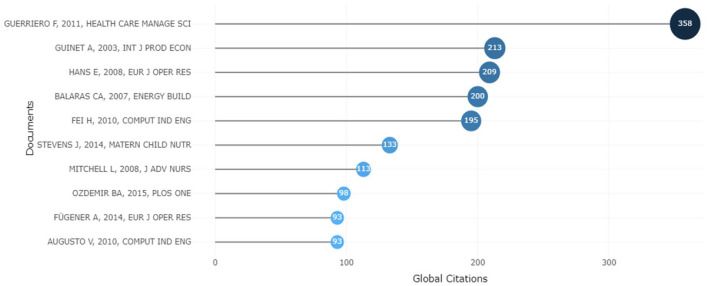
Top 10 globally cited ORS documents from 2000 to 2023.

Biblioshiny was used to conduct historical direct citation analysis regarding local and global direct citations, as shown in Figures 6 and 7. The local citation score refers to the number of articles within a local set of articles that make a reference to a specific article. The 260 documents used in this study comprised the local set. Therefore, the local citation score is inversely correlated with the significance of an article in the ORS research field. On the other hand, the number of articles from the entire Scopus database that mention a certain article is known as the global citation score. Examining historical direct citations, both local and global, provides information on where ORS research is now.

In Figure 6, the horizontal axis represents the local citations, and the vertical axis represents the documents. This figure outlines the top 10 most locally cited documents over the period 2000 to 2023. For example, Guerriero and Guido^49^ is positioned in the highest, and the article was published in the renowned journal “Health Care Management Science (2011).” The article’s major goal is to present a well-organized assessment of the literature on the use of operational research in the scheduling and planning of surgical procedures. Guinet^50^ led the second highest local citations, and the article was published in the renowned journal “International Journal of Production Economics (2003).”; This study mainly focuses on a generic assignment problem of operating room planning. An assignment model with resource capacity and time-window constraints is suggested to handle this problem heuristically. Hans et al^51^ hold the third position in terms of local citation, and the article was published in the renowned European Journal of Operational Research. In order to minimize the needed slack, this research offers a variety of constructive heuristics and local search techniques that employ statistical data on operation durations to exploit the portfolio effect. Moreover, they cited the most previous articles published before them, and they were cited by articles published next to them. Agnetis et al,^52^ Augusto et al,^53^ and Mitchell et al^41^ had the lowest number of local citations. If we consider the last 15 years, the outcome has changed. Aringhieri et al^54^ emerges as the most cited, and Cardoen et al^55^ takes the second position in terms of local citations. Meskens et al^56^ holds the third position in terms of local citations, and the article was published in the renowned journal “Decision Support Systems (2013).” This chapter focuses on the approaches offered and how they address new and evolving practical issues in these domains. Cardoen et al^55^ proposed a combinatorial optimization problem, thereby suggesting a dynamic programing algorithm for pricing issues and column generation as a solution approach.

Similarly, Figure 7 depicts the top 10 most globally cited documents for the period 2000 to 2023. The top 3 articles were the same as those with the highest number of local citations.^49-51^ If we further filter the time span between 2008 and 2023 to search for global citations within the last 15 years, the results change, as Twinanda et al^57^ is positioned at a high value. Cardoen et al^58^ is the second-highest global citation per document. The article was published in the renowned journals IEEE Transactions on Medical Imaging (2017) and the International Journal of Production Economics (2009). For example, Twinanda et al^57^ provided a novel approach to phase recognition that relies solely on visual data and automatically learns features from cholecystectomy movies using a Convolutional Neural Network (CNN). Meanwhile, Cardoen et al^58^ created mixed-integer linear programing solution techniques to aid the operating room scheduler’s decision-making process.

**Figure 8.**
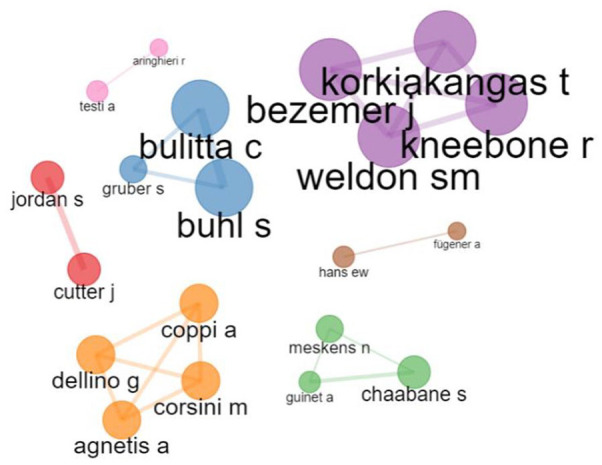
Collaborative networks of leading authors in ORS research (2000-2023).

Figure 8 shows the collaborative citation network, indicating the authors’ collaboration in the operating room scheduling and optimization field. The highest collaboration networks occurred among Korkiakangas, Weldon, Bezemer et al, and Kneebone.^59-61^ Collaborative teams focused on music, communication, learning, and video analysis of communication and awareness in the operating room. The collaboration networks also happened between Aringhieri et al^54^; between, Fei, Chu^62,63^; between Agnetis, Coppi, Pranzo^52,64^; between Fugener and Hans.^51,65^ When it was further filtered between the time span 2008 to 2023 to capture the documents within the last 15 years, we obtained the following results: the authors Galata and Dodaro^66,67^ lead the first position, the authors Toub, Achchab, and Souissi^68-70^ hold the second position, and the third highest collaboration is held among the authors Cardoen, Demeulemeester, and Belien.^25,33,55,58^ In contrast to a recent one, Galata and Dodaro et al^66,67^ described the solution for the problem based on Answer Set Programing (ASP) and explicitly considered bed management.

#### Co-citations & Co-occurrence network

**Figure 9.**
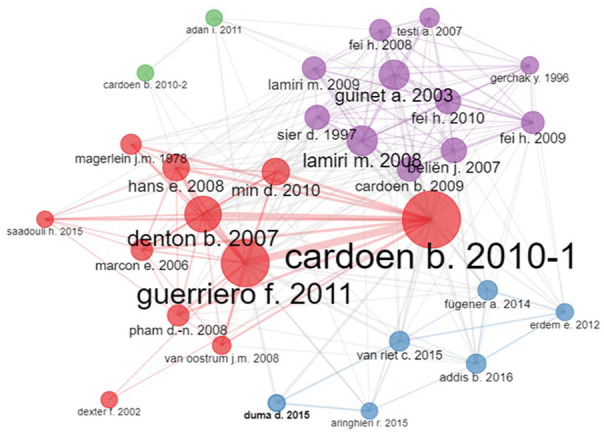
Co-citation network of key ORS publications (2000-2023).

The diversity of journals containing reviewed publications underscores the significance of this topic. As shown in Figure 9, publications are connected by lines to show that other publications have cited them.^71^ Co-citation is a term used in bibliometric analysis to describe citation interaction. The thickness of a line reflects the number of co-citations, which is inversely related to the relevance of the article. The high network density indicates the broad coverage of ORS by numerous OM scientific sources and the commonality of the OR-related content in these articles.^72^ For instance, Guerriero and Guido,^49^ Guinet,^50^ and Hans et al.^51^ was the highest-cited author in terms of local and global citations, as indicated in Figure 7. However, in terms of co-citation, Cardoen et al^33^ and Guerriero and Guido^49^ and Denton et al^73^ were the highest co-cited authors, as shown in Figure 9. Upon refining the data to focus on the period from 2008 to 2023, emphasizing optimization-oriented reviews, Aringhieri^54^ and Cardoen et al^33^ stand out in direct citations. However, in co-citations, Cardoen et al^33^ held the top position. The authors focused mostly on optimization, such as the multi-objective case sequencing problems, decision support systems for cyclic surgery scheduling, data-driven robust optimization, and sequencing surgical cases in day-care environments.

**Figure 10.**
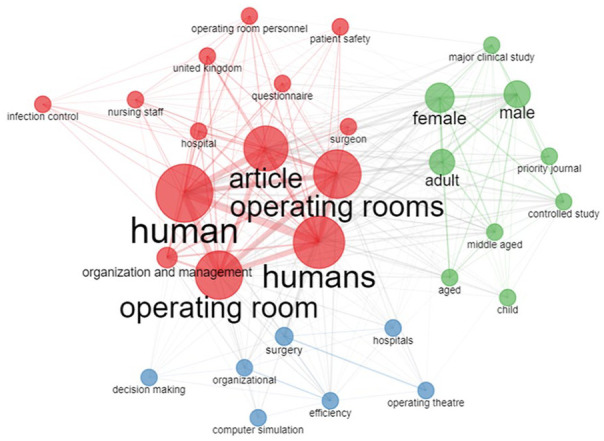
Keyword co-occurrence network in ORS research.

The co-occurrence network, as depicted in Figure 10, indicates that most research revolves around operating rooms, humans, surgery, organization, and management within diverse dimensions of healthcare management. The same filtering technique was adopted for the co-occurrence network, and it was found that most research was based on ORS, surgery, and optimization.

### Analysis of developments and trends in ORS research

These keywords serve as an article’s synopsis. ORS publications’ specialization and research progress can be rapidly perceived using clustering and multiple correspondence analyses. The data mining and statistical analysis capabilities of Biblioshiny are used to carry out these analyses in the subsections.

#### High-frequency keywords

**Figure 11.**
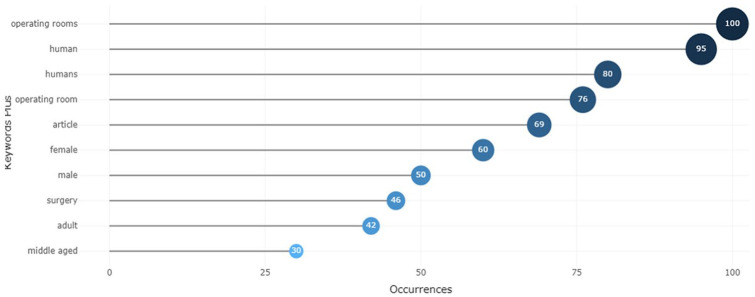
Keyword frequency distribution in ORS publications (2000-2023).

Based on a collection of publications, keyword analysis can characterize and depict the structure of a scientific area.^74^ The authors used keywords to convey the primary ideas in their articles. Using Biblioshiny’s data mining and visualization tools, we created the Pareto diagram shown in Figure 11 to summarize the subjects covered in the examined articles. Operating rooms/operating room, human/humans, male/female, and surgery are the most frequent words, occurring 100/76, 95/80, 60/50, and 46 times in the documents, respectively. The authors further performed filtering for the last 15 years to better capture optimization-oriented operating room scheduling. Operating Rooms, Scheduling and Optimization, which are the main topics of this research, were observed to have the highest frequencies.^20,22,24,30-32,34,35,45,75-81^

Integer programing is the most widely employed optimization model type because of the 3 primary ORS issues of choosing patient treatment days, deciding on patient time slots throughout the day, and allocating patient resources. Researchers can also see the words “Humans, Surgery, Combinatorial Optimization, Efficiency, Simulation” in the previous literature.^40,51,82-87^ The authors also performed forward and backward reference searches to find the most frequent words and found that Operating Room, Surgery, Scheduling, Human, Uncertainty, and Optimization were the most frequent keywords. For example, Rahimi and Gandomi^88^ conducted a comprehensive review of the operating room and surgery scheduling problems and Harris and Claudio^89^ highlighted the recent trends in ORS research between 2015 and 2022 in their review.

**Figure 12.**
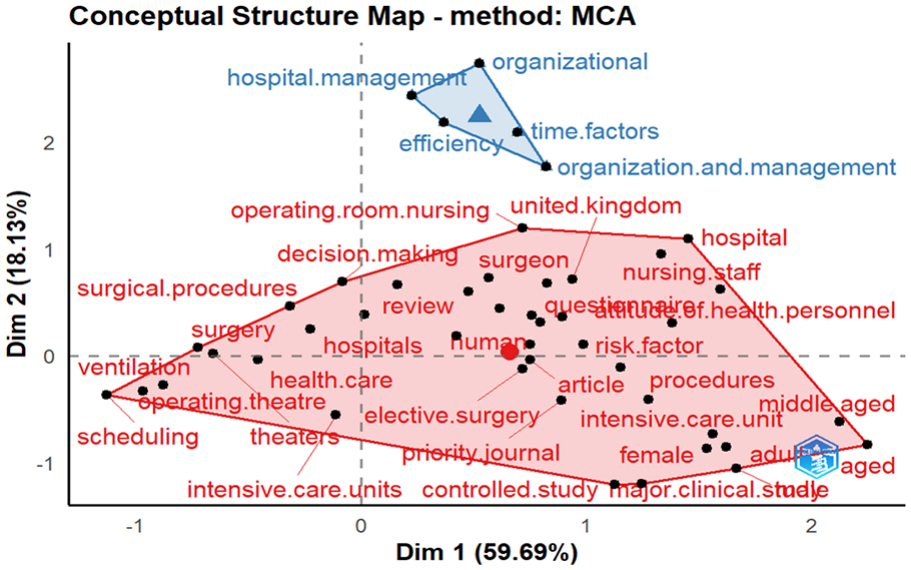
Multiple correspondence analysis (MCA) of keyword clusters in ORS research.

Clustering and multiple correspondence. Keywords in bibliometric data often appear in pairs, and their frequencies can be assessed using cluster analysis. This method uses statistical techniques to simplify an intricate network of keyword relationships into more manageable subnetworks.^90^ On the other hand, Multiple Correspondence Analysis (MCA) is a statistical approach for analyzing and visualizing relationships in multidimensional categorical data, frequently employed with bibliographic data. In the context of an MCA-generated conceptual structure map, “dim 1” refers to the primary dimension that captures the main pattern in the data, and “dim 2” indicates the secondary dimension that highlights a distinct pattern. These dimensions help identify the relationships between bibliographic items, allowing for deeper insights into the underlying data structure. MCA distils a vast array of keywords into a 2- or 3D space, showcasing the similarities between terms. As shown in Figure 12, the similarity of the 2 keywords increases as their distance decreases, and vice versa. The keywords closer to the center of each colored area, which contains each sub-network, have been used more frequently in previous years. Studies in some subnetworks have paid less attention to keywords that are closer to the edges.^91^ Two major clusters are evident in the multiple correspondence diagrams, as shown in Figure 12.

The red cluster covers larger topics, such as operating theater/room, hospitals, decision-making, surgical procedures, elective surgery, health care, risk factors, and human.^73,79,86,92-96^ The authors mainly focused on decision support systems in healthcare surgery and hospital operating room scheduling. They also emphasized that risk factors can affect humans during pre- and post-operative care. Between 2008 and 2023, the 2 major clusters were hospitals, assignment problems, surgical procedures, decision-making, and multi-objective optimization.^20,22,24,30-32,34,35,45,75-80^

The blue cluster covers hospital management, organization and management, efficiency, time factors, and organizational performance.^40,84,85,96-98^ Between 2008 and 2023, the cluster covered large topics, such as operating room, operation duration, humans, hospital management, appointments and schedules, efficiency, and process optimization.^40,51,82-87^

### ORS research evolution analysis

**Figure 13.**
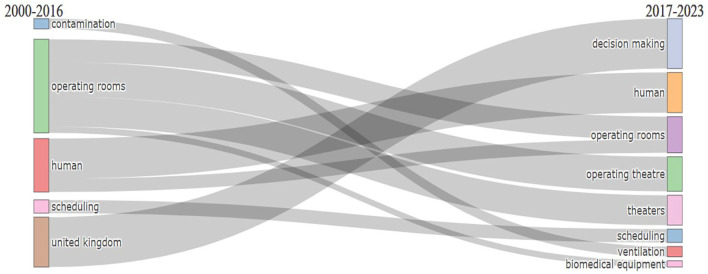
Thematic evolution of ORS research from 2000 to 2023.

The evolution of ORS research themes over time is visually represented using a Sankey diagram, as shown in Figure 13. A Sankey diagram is sometimes known as a Sankey energy difference diagram. Typically, this type of diagram represents financial, material, and energy data. This diagram also discusses quantitative data on thematic flows, orientations, and relationships^99^ and depicts the various themes in ORS flows.

In Figure 13, the topics are shown in rectangles. The number of keywords contained within a theme determines the size of the rectangle. The rectangles were connected by lines depicting the evolution of the study theme. The rectangles’ connections signify a theme’s chronological continuity between nearby time zones. The relationship between topics is indicated by the thickness of a line, which is proportional to the number of shared keywords. Each line’s color was used to distinguish between the study themes. Between 2000 and 2016, researchers focused mostly on the keywords operating rooms, humans, scheduling, and contamination. However, between 2017 and 2023, researchers focused mainly on the keywords decision-making, human, operating room, scheduling, and operating theater.^1,32,75,81,100-105^

Upon applying the aforementioned filtering criteria, it was observed that between 2008 and 2018, the primary research keywords were operating rooms and humans, followed by the Monte Carlo method and healthcare. In contrast, from 2018 to 2023, the research emphasis was on efficiency and scheduling, followed by heuristic methods, optimization, and humans.^21,23,43,70,81,106,107^ A consistent observation across these time spans is the central focus on humans.

The thematic evolution map in Figure 13 provides a comprehensive overview of the ORS research journey, highlighting the themes’ emergence, evolution, and potential extinction. It also showcases the dynamic nature of ORS research, evident from the diverse research topics across different timeframes and the intricate web of thematic relationships. The observed patterns of theme amalgamation, transition, and rejuvenation further underscore the evolving landscape of ORS research.

**Figure 14.**
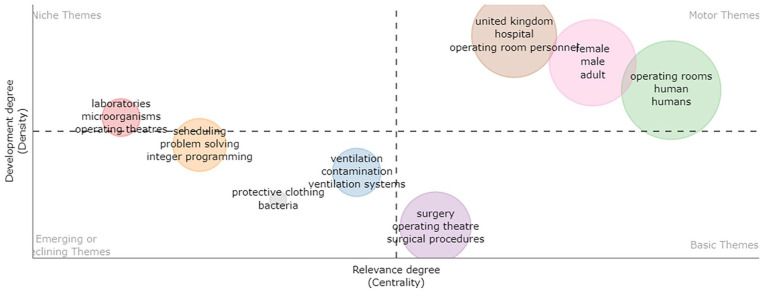
Thematic centrality and evolution in ORS research: 2000 to 2023.

The centrality and evolution of themes in ORS research are depicted in Figure 14. Historically, ventilation systems and contamination have been central themes, complemented by foundational topics such as operating rooms/theaters, surgical procedures, and surgery.^62,63,108,109^ As the field has progressed, emerging themes, such as protective clothing, bacteria, ventilation systems, and integer programing, have come to the forefront.^1,109-111^ Niche themes such as laboratories and microorganisms have found unique spaces in the research landscape.

Contamination, bacteria, ventilation systems, and protective clothing in the operating room are discussed in details under *Contamination, bacteria, ventilation systems, and protective clothing in the operating room* Section. The motor themes include operating rooms, humans, and hospitals; this research is also rapidly growing in the United Kingdom. The development of evolutionary stages focuses on hospitals, AI, and multi-objective optimization. Between 2008 and 2023, It has shown that the human and operating rooms maintained centrality, followed by basic themes such as operating rooms, scheduling, and surgery. An emerging theme is the use of neural networks. Niche themes include column generation and computational efficiency; this research is rapidly growing in Belgium. The development of evolutionary stages focuses on hospitals, AI, and multi-objective optimization.

In conclusion, the primary aim of our study is to comprehensively assess the landscape of ORS through a bibliometric analysis. We intend to convey a clinical message emphasizing the need for an integrated approach in ORS, aligning with healthcare objectives for improved patient outcomes, economic efficiency, and value-based care. The identified research trajectories, encompassing Surgery Duration, Allocation, Advanced Scheduling Integration, and Patient Flow Optimization, provide valuable insights for practitioners and policymakers. Furthermore, our bibliometric findings highlight the prominence of heuristic algorithms and integer programing, suggesting their importance in optimizing OR scheduling for enhanced healthcare delivery.

## Limitations and Future Research Directions

Although this study offers significant insights, there are certain limitations to consider. Primarily, our reliance on Scopus as the sole database for literature extraction may have narrowed the scope. Although Scopus is considered the largest abstract and citation database of peer-reviewed literature, overlapping documents exist in other databases, such as the Web of Science, ProQuest, and PubMed. To capture a more holistic view, especially encompassing the social, behavioral, or clinical facets of operating room scheduling, diversifying the databases could provide a richer literature review.

Another limitation is the emphasis on English-language articles, inadvertently sidelining research in other languages, such as Japanese and Spanish. This could mean that certain ORS challenges addressed in these languages remained unexplored in our study. Our search was also confined to the “Title-abstract-keywords” option in Scopus, which might have restricted the breadth of the results. Many of the 260 items retrieved were tangentially related or unrelated to the core topic. Given the multidisciplinary nature of the ORS, we aimed to maintain the focus of the study and exclude less relevant publications.

Transitioning from these limitations, it is evident that there is a vast landscape of opportunities for future research in the realm of ORS. Future studies could benefit from more exhaustive bibliographic or mapping analyses, such as bibliographic coupling and co-citations of cited authors and journals. A deeper dive into the identified clusters and themes would enrich the research landscape and pave the way for subsequent investigation. As the field evolves, monitoring shifts in the conceptual framework and the primary research questions is crucial.^112^ The fundamental uncertainties in surgical services, particularly stochastic surgical durations and their scheduling implications, warrant more attention. Emphasizing the integration of models that address these uncertainties is paramount,^113^ especially given that only half of recent studies have addressed such integration.^81^ Recent trends in ORS research have highlighted the use of multi-criteria decision-making tools for optimization under uncertainty and heuristic methods. Emerging topics include concerns about contamination, bacteria, and the importance of ventilation systems and protective clothing in operating rooms. Some researchers have also explored priority rules such as First-In-First-Out (FIFO), Earliest Due Date (EDD), and Slack Time Remaining (STR) for scheduling in flexible job shop systems. A review of the current literature underscores the fact that ORS research is still nascent. Potential avenues for future research include comprehensive optimization studies, performance metrics, generic models, Industry 4.0 & 5.0 implications, and the application of Multi-Criteria Decision-Making Methods (MCDM) in ORS. This represents the evolving trajectory and emerging trends in ORS research.

### Robotic and computer-assisted surgery

Robotic surgery is a new, cutting-edge technology that has revolutionized the surgical field. This revolution fundamentally altered surgical practices through the integration of numerous technological advancements. The eyes and hands of the surgeon are replaced by high-definition cameras and micro-instruments, which enter the body through tiny incisions. Innovations in robotics and computer science have improved surgeons’ ability to perform difficult surgeries accurately and precisely. Surgeons have access to instruments that grant abilities that can substitute the lack of physical probing to identify target structures, surgical planes, and resection margins, similar to aeronautics and military equipment. Many challenges and drawbacks will eventually be overcome, and several new inquiries will undoubtedly arise. Questions such as malpractice responsibility, credentials, training standards, and interstate licensure for telesurgeons, to name just a few, have yet to be raised.^114,115^

### Contamination, bacteria, ventilation systems, and protective clothing in the operating room

A hospital’s OR is a unique area that needs to be kept clean. The rates of surgical site infections are extrinsically influenced by the microbial population of an indoor OR,^116^ and cleanliness of the air is essential in operating rooms.^117^ The patient, surgical team, surgical equipment, and operating environment are sources of contamination in the operating room or theater. Utilizing PPE (gloves and yellow gowns) for direct patient contact or when transferring patients is the most efficient strategy to reduce this contamination. Before leaving the patient’s room, care area, or OR, the surgical team members should always take off their PPE. After removing the protective equipment, the surgical team should wash their hands thoroughly with soap and water to avoid exposure to feces from diarrhea.^116,117^

In healthcare facilities, effective ventilation systems can maintain low levels of airborne particles and microbial air contamination, lower the risk of infection through airborne transmission, and guarantee healthy and secure working conditions for medical staff. The novel Coronavirus COVID-19 emergency has made surgical staff in the operating room and on the outside more conscious of virus transmission.^118,119^ The impacts of surgical procedures, operating equipment, ventilation systems, surgical gowning systems, and microbiological contamination should be investigated in the future through a new broad experimental campaign.

### Music and communication in the operating theater

The integration of music within the operating theater is a multifaceted consideration in surgical practice, bearing significance not only on the ambient environment but also on communication and team dynamics. For instance, about 53% to 72% of surgical procedures use music.^59^ Research demonstrates that music can reduce stress, elevate team morale, and improve communication within surgical teams. Future research directions should explore how music contributes to a calmer working atmosphere, positively influences teamwork, and enhances the patient experience. By acknowledging diverse practices and citing relevant literature, the section underscores the interdisciplinary nature of healthcare. It establishes the importance of considering music as a factor influencing communication and contributing to the overall well-being of individuals in the operating theater.^120^ The operating room was already louder than the World Health Organization recommended. There is considerable disagreement over whether music should be played in operating rooms, even though little research has been conducted and no guidelines or rules have been established. Although it is often overlooked as a possible safety threat, music performed in the operating room can hinder team communication. The choice of music, its volume, and whether music is played are mostly decisions made by surgeons. For recommendations and guidance, open dialog between clinicians, managers, patients, and governing authorities should be encouraged.^59,120^

### Data analytics and artificial intelligence (AI) in operating room/theater

AI research in surgery is growing quickly, covering topics such as image recognition in the operating theater to ML algorithms’ capacity to better anticipate the risk of surgery a priori and influence decision-making. AI may lead to new surgical advancements that can change how the procedures are performed. ML models are particularly effective when used with large volumes of source data or “big data” and can be applied to nearly any field where pattern detection is valuable. Many ML models have been used in clinical medicine to estimate surgery duration.^37,121^ Black box and interpretable models are 2 general types of models emphasized in some research. This highlights one instance of each to highlight its potential clinical applications in surgery. AI will play a significant role in the future of surgery and medicine in several areas, including patient-targeted interdisciplinary care, screening, diagnosis, and therapy^122,123^

### Ethical and legal considerations

Future research should address ethical and legal issues because operating room scheduling involves sensitive patient data and important judgment calls. Assuring patient privacy and data protection, addressing scheduling concerns for fairness and equity, and adhering to legal obligations were all included in this study.

### Uncertainty and risk management

Operating room scheduling is inherently uncertain due to variables including surgery length, patient state, and emergent cases. Future studies could investigate techniques such as stochastic programing, resilient optimization, and scenario-based modeling to manage risk and uncertainty in scheduling. These methods can aid in creating more adaptable and resilient scheduling plans.

### Collaborative scheduling and resource sharing

Collaborative scheduling techniques can promote coordination and resource sharing between various surgical centers and hospitals. Research can investigate the creation of scheduling frameworks and models that promote institutional cooperation and resource sharing, resulting in better resource use and shorter patient waiting times.

### Telemedicine and virtual reality integration

The integration of Telemedicine and Virtual Reality (VR) in the operating room represents a cutting-edge advancement in healthcare technology. With the development of Telemidicine and virtual reality technologies, it may be possible to include these technologies in the planning of operating rooms. When combined with virtual reality, which creates immersive simulated environments, this integration offers a range of benefits in the operating room setting. Surgeons can utilize VR for pre-operative planning, training, and simulation of complex procedures. Telemedicine enables remote expert consultation during surgeries, fostering collaboration and knowledge exchange. Together, these technologies enhance accessibility to specialized care, improve surgical precision, and contribute to the evolution of more efficient and collaborative surgical practices.^124,125^

### Green operating room scheduling

Sustainability in health care is becoming increasingly important. Future studies should examine green operating room scheduling strategies that limit energy use, reduce waste generation, and optimize resource usage to increase environmental sustainability in surgical operations.

### Human factors and workflow optimization

Improving scheduling procedures might result from understanding the human factors involved in operating room scheduling. The effectiveness of scheduling can be examined in terms of how human factors such as surgeon preferences, staff workload, and communication dynamics affect them. Furthermore, improving teamwork and coordination across many teams participating in surgical procedures can improve scheduling outcomes.

## Conclusion

A bibliometric study of operating room scheduling literature was conducted based on bibliographical information from the Scopus database. The Biblioshiny program in the Bibliometrix package was used for data mining and analysis. There are numerous traits and focuses of ORS. The number of published ORS articles has consistently increased annually in terms of publication trends. The number of publications has increased significantly since 2013. Consequently, the increase in ORS-related articles during the past 15 years may be broken down into 2 phases: the developing stage (2008-2012), which had a low frequency of publications. In the last step, which spanned 2013 to 2023, there were the most papers on ORS research. There has been a noticeable rise in researchers’ involvement and interest in ORS research. Fhoula et al conducted a bibliometric and graphical study to examine the status and structure of the ORS field’s knowledge domains (social, intellectual, and conceptual networks). Before outlining the main contributions, we first examined the overall patterns of published articles while considering various nations, organizations, publications, and authors. Additionally, we analyzed the major study themes using co-occurrence analysis and discovered fundamental literature using co-citation analysis.^112^

Developed nations like the United Kingdom, Australia, the United States, France, and Germany have predominantly spearheaded ORS research. Key terminologies, such as operating rooms, humans, males/females, and surgery, emerged as recurring themes. Between 2008 and 2023, countries like China, the United States, Belgium, and Germany were at the forefront, with North America and Australia comparatively less active. The overarching themes during this period were “Operating Rooms” and “Optimization. The consistent emphasis on 'Human' and 'Operating Room Scheduling” from 2000 to 2023 underscores their centrality in ORS research. This study identified 7 pivotal research trajectories in ORS, encompassing diverse areas, such as Surgery Duration, Allocation, Advanced Scheduling Integration, and Patient Flow Optimization.

Operating room scheduling utilizing heuristic algorithms and integer programing is the topic of highly cited OR publications in the Scopus database. These highly referenced articles demonstrate a variety of research streams in ORs. The results of this study will aid practitioners and policymakers in making well-informed decisions on future ORS problems, as well as academics in this field of study, to understand the present state of knowledge required to advance their research.

### Academic and practical implications

This study comprehensively examines the landscape of Operating Room Scheduling (ORS) research, spanning 2000 to 2023, and harnesses advanced bibliometric methodologies. Its contributions are diverse and resonate in both academic and practical arenas. From an academic standpoint, it illuminates chronological progression, contemporary trends, and prospective research trajectories in ORS. This serves as an invaluable compass for scholars, researchers, and academicians in healthcare management and operations research, spotlighting pivotal research themes and uncharted territories. This roadmap fosters innovation and encourages cross-disciplinary synergies. In the practical spectrum, these implications are profound. Healthcare policymakers, administrators, and frontline practitioners can connect these insights to recalibrate resource distribution, amplify operational prowess, and elevate patient-centric care in operating rooms. Furthermore, the study underscores the potential of leveraging technology and data analytics in ORS. By integrating predictive analytics, institutions can anticipate surgical delays, optimize resource allocation, and enhance patient outcomes. The emphasis on global perspectives also paves the way for transnational collaborations, fostering best-practice exchanges and benchmarking. With its depth and breadth, this research is a cornerstone for propelling ORS forward and catalyzing improvements in healthcare delivery and patient experiences.

Examining the findings in the clinical context reveals a substantial impact on operational efficiency and patient-centric outcomes in healthcare. As highlighted by our bibliometric analysis, the emphasis on an integrated approach in ORS aligns with the overarching goals of clinical practice. The identified research trajectories, particularly in Surgery Duration, Allocation, Advanced Scheduling Integration, and Patient Flow Optimization, directly influence the operational dynamics within clinical settings. Practical insights drawn from these trajectories hold the potential to optimize surgical processes, reduce waiting times, and enhance resource allocation, thereby contributing to improved patient outcomes. Furthermore, the recognized significance of heuristic algorithms and integer programing underscores their relevance in clinical decision-making for OR scheduling, suggesting their pivotal role in streamlining processes and promoting efficiency in healthcare delivery. Overall, the clinical implications of our study underscore a pathway toward more effective and patient-centric OR management strategies.
